# Intermittent energy restriction vs. continuous energy restriction on cardiometabolic risk factors in patients with metabolic syndrome: a meta-analysis and systematic review

**DOI:** 10.3389/fnut.2023.1090792

**Published:** 2023-05-09

**Authors:** Rui Xu, Youxiang Cao, Peng-Ying Wang, Xiao-Lan Chen, Dan Tao

**Affiliations:** ^1^School of Sports and Health, Nanjing Sport Institute, Nanjing, China; ^2^Sports and Health Engineering Collaborative Innovation Center of Jiangsu Province, Nanjing, China; ^3^School of Kinesiology, Shanghai University of Sport, Shanghai, China; ^4^Department of Government and International Studies, Hong Kong Baptist University, Hong Kong, Hong Kong SAR, China

**Keywords:** metabolic syndrome, intermittent energy restriction, continuous energy restriction, obesity/overweight, meta-analysis

## Abstract

**Background:**

This is a systematic review and meta-analysis to compare the efficacy of intermittent energy restriction (IER) vs. continuous energy restriction (CER) on weight loss, body composition, blood pressure, and other cardiometabolic risk factors in patients with metabolic syndrome (MetS) risk factors.

**Methods:**

We searched and screened PubMed, Embase, Cochrane Library, and Web of Science from inception to May 8, 2022 for randomized controlled trials. Two review authors independently selected studies, extracted data, assessed quality and risk of bias and cross-checked extracts to resolve discrepancies when required. We expressed effect size as mean difference (MD) and 95% confidence interval (CI). The major outcome was the improvement of MetS risk factors, including changes in waist circumference (WC), triglycerides (TG), high-density lipoprotein cholesterol (HDL-c), blood pressure (BP), and fasting plasma glucose (FPG) levels. The secondary outcomes were body weight (BW), body mass index (BMI), body fat (BF), fat free mass (FFM), hip circumference (HC), fasting insulin (FINs), total cholesterol (TC), and low-density lipoprotein cholesterol (LDL-c).

**Results:**

The meta-analysis included 16 articles (20 trials) with a total of 1,511 participants. All studies had a low risk of bias for random sequence generation. The IER and CER intervention equally improved MetS risk factors WC (MD = −0.47, 95% CI [−1.19, 0.25]), TG (MD = −0.02 mmol/L, 95% CI [−0.11, 0.07]), FPG (MD = −0.02 mmol/L, 95% CI [−0.10, 0.05]) and BP (systolic blood pressure: MD = 0.93 mmHg, 95% CI [−2.74, 4.61]; diastolic blood pressure: MD =1.15 mmHg, 95% CI [−0.24, 2.55]), but HDL-c (MD = 0.03 mmol/L, 95% CI [0.01, 0.05]) was significant improved in IER when compared with CER. For second outcomes, BW (MD = −0.8 kg, 95% CI [−1.26, −0.33]), BF (MD = −0.75 kg, 95% CI [−1.73, −0.13]) and FFM (MD = −0.49 kg, 95% CI [−0.92, −0.05]) were also significant improved in IER, and not for other outcomes.

**Conclusion:**

Both IER and CER could improve MetS biomarkers, but IER was more effective than CER in the improvement of HDL-c only. For secondary outcomes, IER was also more effective for BW, BF and FFM, but there were no differences in effects for other outcomes.

## 1. Introduction

With the global obesity epidemic, more than 100 million children around the world are obese. This important health concern (1) has been closely associated with type 2 diabetes, coronary heart disease, cancer, and metabolic syndrome (MetS). Especially in the case of MetS, obesity is a major risk factor among the younger generation (2–4). MetS is characterized by multidimensional needs and simultaneously meets multiple indicators. The MetS constellation includes not only obesity but also glucose intolerance, hypertension, and dyslipidemia, which are associated with complications and a risk factor for insulin resistance (type 2 diabetes), hypertension, hyperlipidemia (5, 6), and cardiovascular disease (7).

Lifestyle changes like physical activity, energy restrictions as the best way to eliminate the risk factors related to MetS (8, 9). Energy restriction feeding is a calorie restriction strategy for weight loss that protects against diet-induced obesity and improves glycolipid metabolism (10–12). In many papers, it has been shown that restriction diets have a beneficial effect on systolic or diastolic pressure, lipid profile, glucose homeostasis, body weight, inflammation process, fat mass (13). The continuous energy restriction (CER) diet is a traditional daily calorie restriction method for reducing energy intake by a small amount (e.g., 25–30%) each day (14, 15). The intermittent energy restriction (IER) diet has recently been proposed as an appealing nutritional strategy for obesity management, involving extended time-restricted feeding periods (e.g., 16–48 h) with little or no energy intake, with intervening periods of *ad libitum* feeding, intermittent fasting with little energy intake (e.g., 25–30%) on alternate days, or the 5:2 diet, which includes 5 days of a normal diet pattern, 2 days of fasting 2 days per week, and *ad libitum* intake for another 5 days (16–18).

Both IER and CER have recently received considerable interest as dietary restriction strategies for weight loss and improving glucose and lipid metabolism (10–12, 19, 20). However, their effectiveness is controversial. Previous researchers have reported that IER and CER have an equivalent effect on weight loss (21), but they found different effects on body composition (22–24) and glycolipid metabolism (11, 24). Mechanistically, their energy deficit was not equal in the CER and IER comparisons, and the efficacy of IER vs. CER on weight loss or improving glucolipid metabolism disorder in obesity and MetS remained controversial.

Several reviews and meta-analyses have shown that the effect of IER and CER on weight loss, lipid profile, and glucose intolerance in participants with obesity or type 2 diabetes was comparable (25–27). However, the studies they include were not based exclusively on IER and CER interventions for comparison; some meta-analyses included only the IER intervention. Furthermore, the studies were not entirely focused on MetS. Therefore, in this study we aim to compare the effectiveness of IER and CER regarding MetS.

## 2. Methods

We performed this systematic review according to the Preferred Reporting Items for Systematic Reviews and Meta-Analyses (PRISMA) statement (28). Study protocol was registered on PROSPERO (CRD42023397188).

### 2.1. PICO questions

The search keywords were operationalized using a Population, Intervention, Comparison, Outcome (PICO) chart. Population: adults with MetS risk factors; Intervention: IER and CER interventions; Comparison: IER vs. CER; Outcome: WC, TG, HDL-c, BP, and FPG levels as primary outcomes and BMI, BF, FFM, HC, fasting insulin (FINs), TC, and LDL-c were the secondary outcomes.

### 2.2. Search strategy

We searched electronic databases (PubMed, Embase, Cochrane Library, and Web of Science) to compile our data, with a publication deadline of May 8, 2022. Two sets of key words and their main subtitles to conduct our data search. These sets included calorie restriction (“intermittent fasting,” “intermittent energy restriction,” “alternate day fasting,” “time-restricted feeding,” and “intermittent caloric restriction”) and metabolic syndrome (“obese,” “obesity,” “overweight,” “adiposity,” “metabolic syndrome,” “metabolic disease” and “syndrome X”). The detailed search strategy is shown in Supplementary material. We limited the searched articles to those written in English.

### 2.3. Inclusion and exclusion criteria

Studies were eligible if they met the following criteria: (a) The participants were adult patients (aged > 18 years) with MetS (29); (b) Randomized controlled trials (RCTs) or parallel study designs, excluding uncontrolled trials, conference summaries, observational studies, matched controlled trial designs, and animal studies; (c) Intervention lasting = 2 weeks, with IER and CER measures implemented in experimental groups. The IER protocols involved the period restriction pattern, which included 5:2 or 4:3 IER modes (fasting 2 or 3 days per week and habitually dieting for the remaining days), or alternate-day fasting. The CER protocols involved restricting 20–30% of the necessary daily calories; (d) Reporting of the change in body composition, waist circumference (WC), TGs, HDL-c, BP, and FPG; (e) Studies published in English; (f) Subjects of original articles > 10; and (g) Containing at least one of the following outcomes: BW, BMI, FFM, or BF.

Exclusion criteria were as follows: (a) Studies with unreliable designs or substantial statistical errors; (b) Only one type of diet regimen included; and (c) Inability to access the full text. Two researchers independently selected the literature after screening and evaluation of the selected articles.

### 2.4. Data extraction

Two researchers extracted data and research feature information from qualifying literature (XU, CAO), and a third discussed inconsistencies and settled disagreements. They excluded duplicate studies from the different search engines.

The extraction content of the literature included the following: first author, publication year, country (region), characteristics of participants (sample size, age, sex), intervention characteristics, and outcomes. Repeat studies for the same trials were only included once. We compared IER with CER, without restriction on the treatment history.

The major outcome was the improvement of MetS, including changes in WC, TG, HDL-c, BP, and FPG levels. The secondary outcomes were BMI, BF, FFM, HC, fasting insulin (FINs), TC, and LDL-c.

### 2.5. Assessment of risk of bias

Two researchers independently assessed the risk of bias using the Cochrane risk-of-bias tool (30), which contains 7 domains of bias: (i) random sequence generation; (ii) allocation concealment; (iii) blinding of participants and personnel; (iv) blinding of outcome assessment; (v) incomplete outcome data; (vi) selective reporting; and (vii) other biases. The researchers evaluated each domain with “high risk,” “low risk,” and “unclear” indicators.

### 2.6. Assessment of heterogeneity

We analyzed the heterogeneity between trials by means of the χ^2^ test, and they regarded a *P*-value of <0.1 as indicating significant heterogeneity. We computed Cochran’s Q statistic by using the following formula to calculate the *I*^2^ statistic, which we used to qualify heterogeneity because it does not depend on the number of studies (31). The magnitude of heterogeneity was categorized as follows: *I*^2^ of 0–24% being low, *I*^2^ of 25–49% being moderate, *I*^2^ of 50–74% being substantial, and *I*^2^ of 75–100% being high (31).


(1) $$\left[{\text{I}}^{2}\,=\,\frac{\text{Q}-\text{df}}{Q}\right]$$

where df denotes the degree of freedom, obtained by subtracting 1 from the number of trials.

If moderate or high heterogeneity existed between the studies, we used the random effect model; otherwise, we adopted the fixed-effect model. We also conducted a sensitivity analysis by changing the pooled model or by adopting a 1 × 1 exclusion approach for moderate or high heterogeneity.

### 2.7. Data synthesis and analysis

We performed data synthesis with Statistics and Data Science (Version 16.0; Stata Corporation) and Review Manager (Version 5.3; Copenhagen, Denmark: The Nordic Cochrane Centre, The Cochrane Collaboration). We presented the effect of the intervention as mean difference (MD) or standardized mean difference (SMD) as effect analysis statistics before and after intervention. The MD and 95% CI were calculated for the effect size between IER and CER. If statistical heterogeneity existed among the results, we further analyzed the source of heterogeneity.

### 2.8. Safety

Participants were well tolerated in articles and no major adverse events were reported. Minor physical or psychological adverse effects, such as tired, cold intolerance, constipation, hair loss, headaches, mood swings or bad temper, mild cognitive impairments, temporary sleep disturbance were reported in a minority of participants in a few studies (23, 24, 32). These adverse effects were only reported in the intervention phase in most articles, and were resolved when the dietary restriction intervention was terminated.

## 3. Results

### 3.1. Literature search

**FIGURE 1:**
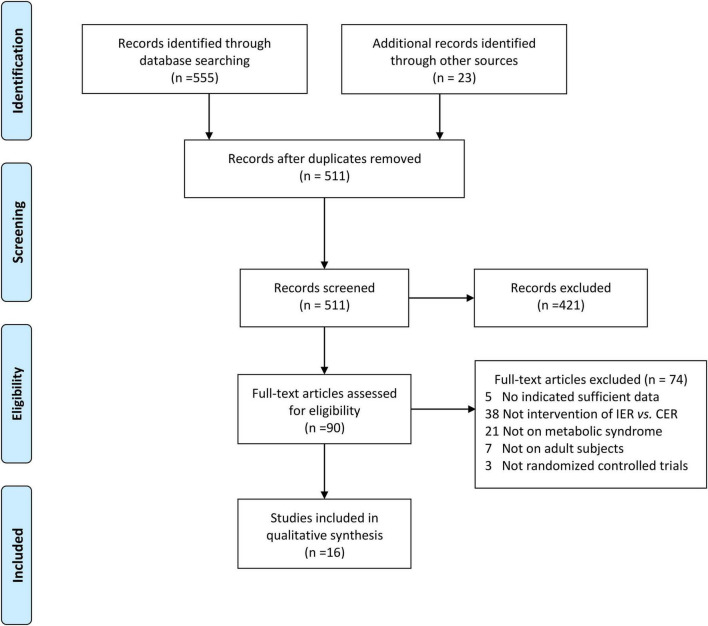
PRISMA flow diagram.

The details of the search are shown in Figure 1. We identified a total of 555 potential studies from PubMed, Embase, Cochrane Library, and Web of Science. Based on the year of publication and the type of study, we included 23 studies. After reexamination, we deleted 67 duplicated studies. After reviewing the titles and abstracts, we excluded 421 non-conforming studies. After reviewing the full texts, we excluded 74 studies that did not meet the requirements. Finally, 16 studies met the selection criteria (11, 22–24, 32–43).

### 3.2. Study characteristics

We included 16 articles (20 trials) with 1,511 participants (Table 1). The characteristics of the studies are presented in Tables 1, 2. The 20 trails were primarily RCTs. The patients were diagnosed with MetS. One trial was on an intermittent diet with a continuous diet, but the authors did not report the diet protocol (35); 9 trials were on 5:2 intermittent energy restriction with CER (11, 23, 24, 32, 36, 38, 39, 41); 4 trials were on 4:3 intermittent energy restriction with CER (22, 40, 43); and one trial was on fasting on alternate days with CER (37). Period energy restriction, 5:2 mode, 4:3 mode, and alternate-day fasting mode are all forms of IER. All studies included data on BW, BMI, and the risk factors or indicators related to MetS. All dietary intervention methods met the standard criteria, and data measurements were ensured to minimize errors. The studies included regular follow-up to ensure accuracy. All subjects were older than 18 years.

**TABLE 1:** Characteristics of included studies.

References	Country	Participants	Outcomes	Age (year)	Female (%)	Study duration	CER intervention detail	IER intervention detail	Sample size (IER/CER)
Wing (33)	United States	Adults with obesity and T2D	➁➃➄	52.3 ± 10.7	64.5	12 months	1000–1200 kcal/day	490–540 kcal/day in 1–12 weeks and 24–36 weeks	IER:38 CER:41
Williams (34)	United States	Overweight/obesity and T2D	➁➂➄	51.4 ± 7.9	61.1	20 weeks	1500–1800 kcal/day	400–600 kcal/day for 5 consecutive days during weeks 2, 7, 12, and 17	IER:18 CER:18
Harvie (11)	United Kingdom	Overweight/obesity	➀➁➄	40 ± 3.9	100	1 months	75% of baseline energy needs daily, based on a Mediterranean type diet	25% of baseline energy needs daily on 2 consecutive days, no restriction on the other 5 days	IER:54 CER:53
Harvie (11)	United Kingdom	Overweight/obesity	➀➁➄	40 ± 3.9	100	3 months	75% of baseline energy needs daily, based on a Mediterranean type diet	25% of baseline energy needs daily on 2 consecutive days, no restriction on the other 5 days	IER:54 CER:53
Harvie (11)	United Kingdom	Overweight/obesity	➀➁➄	40 ± 3.9	100	6 months	75% of baseline energy needs daily, based on a Mediterranean type diet	25% of baseline energy needs daily on 2 consecutive days, no restriction on the other 5 days	IER:54 CER:53
Arguin (35)	Canada	Obesity and post-menopausal	➀➂➃	61 ± 7.3	100	15 weeks	−372 ± 377 kcal/day during continuous diet intervention	−372 ± 377 kcal/day for 5-week energy restriction periods, followed 5-week weight stabilization periods	IER:12 CER:10
Harvie (24)	United States	Overweight	➀➃➄	47.9 ± 7.7	100	1 month	Approximately 6000 kJ/day for 7 day/week	2500–2717 kJ/day for 2 consecutive days and consume a euenergetic Mediterranean-type diet for the remaining 5 day of the week	IER:37 CER:40
Harvie (24)	United States	Overweight	➀➃➄	47.9 ± 7.7	100	3 months	Approximately 6000 kJ/day for 7 day/week	2500–2717 kJ/day, for 2 consecutive days and to consume a euenergetic Mediterranean-type diet for the remaining 5 day of the week	IER:37 CER:40
Carter (36)	Australia	Overweight/obesity and T2D	➀➃➄	62 ± 9.1	52.4	12 weeks	5000–6500 kJ/day	1670–2500 kJ/day for 2 days each week, with the remaining 5 days as habitual eating	IER:26 CER:25
Catenacci (37)	United States	Obesity	➀➁➂	42.7 ± 7.9	76	8 weeks	400 kcal/day deficit from estimated energy requirements	Fast on alternate days, a diet estimated to meet estimated energy requirements	IER:13 CER:12
Antoni (38)	United Kingdom	Overweight/obesity	➀➂➄	48 ± 3	53.8	IER:59 days CER:73 days	A daily hypoenergetic diet of 2510 kJ below their estimated energy requirements	25% of their estimated euenergetic needs, 2 consecutive days of the week, another 5 days consume an euenergetic healthy diet	IER:15 CER:12
Carter (39)	Australia	Overweight/obesity and T2D	➀➃➂➄	61 ± 9.2	56.2	12 months	1200–1500 kcal/day	500–600 kcal/day for non-consecutive 2 days and followed their usual diet for the other 5 days.	IER:51 CER:46
Schübel (23)	Germany	Overweight/obesity	➀➃➄	50.5 ± 8	49	12 weeks	80% of the individual energy requirement daily	25% of the individual energy requirement for non-consecutive 2, 5 days of the week were based on a eucaloric balanced diet	IER:49 CER:49
Sundfør (32)	Norway	Obesity	➀➁➄	47.5 ± 11.6	50	3 months	Consume ∼80% of the individual energy requirement daily	400/600 kcal (female/male) on two non-consecutive days. the weekly average calorie intake corresponded to ∼80% of the normal energy requirement	IER:54 CER:58
Sundfør (32)	Norway	Obesity	➀➁➄	47.5 ± 11.6	50	6 months	Consume ∼80% of the individual energy requirement daily	400/600 kcal (female/male) on two non-consecutive days. the weekly average calorie intake corresponded to ∼80% of the normal energy requirement	IER:54 CER:58
Parvaresh (40)	Iran	Overweight/obesity	➀➁➂	46.4 ± 7.9	40.6	8 weeks	70% of baseline energy needs daily	25% of baseline energy needs daily on 3 non-consecutive days 100% of baseline energy needs daily for 3 non-consecutive days, and 1 *ad libitum* without limitation	IER:35 CER:34
Sundfør (41)	Norway	Obesity	➀➁➂➃	49.8 ± 10.5 46.7 ± 12.1	50	12 weeks	About 26–28% less than calculated needs	Near-fasting (400 kcal/day for females and 600 kcal/day for males) on two non-consecutive days weekly while eating usual on the remaining 5 days a week	IER:50 CER:48
Hutchison (22)	Australia	Overweight	➀➁➃	51 ± 2	100	8 weeks	70% of calculated baseline energy requirements daily	A 24 h fast on three non-consecutive weekdays per week, 100% of energy requirements on fed days	IER:25 CER:26
Maroofi (42)	Iran	Overweight/obesity	➀➁➃	45.2 ± 11.7	71.6	8 weeks	70% of baseline energy needs daily	30% of baseline energy needs daily for 3 days while regular diet for 4 days	IER:44 CER:44
Razavi (43)	Iran	Overweight/obesity	➀➁➃	43.1 ± 9.26	37.3	4 months	75% of baseline energy needs daily	25% of baseline energy needs daily on non-consecutive 3 days while regular diet for 4 days	IER:35 CER:34

CER, continuous energy restriction; IER, intermittent energy restriction. ①WC ②TG ③HDL-c ④SBP and/or DBP ⑤FBP.

**TABLE 2:** Comparison the effects of IER and CER on body weight and body composition.

Outcomes			Within-group		IER vs. CER
			**IER**	**CER**	
BW (kg)	Trails		20	20	20
	MD (95% CI)		−5.46 (−6.85, −4.07)	−4.94 (−6.47, −3.41)	−0.80 (−1.26, −0.33)
	Heterogeneity	*I* ^2^	0	0	0
		*P*	0.94	0.96	0.87
	*P*		<0.001	<0.001	<0.001
BMI	Trails		8	8	8
	MD (95% CI)		−2.35 (−2.96, −1.74)	−2.27 (−2.95, −1.60)	−0.16 (−0.57, 0.24)
	Heterogeneity	*I* ^2^	52	0	50
		*P*	0.04	0.45	0.05
	*P*		<0.001	<0.001	0.42
BF (kg)	Trails		11	11	11
	MD (95% CI)		−4.80 (−6.05, −3.55)	−2.79 (−4.39, −1.19)	−0.75 (−1.73, −0.13)
	Heterogeneity	*I* ^2^	0	0	0
		*P*	0.98	1.00	0.52
	*P*		<0.001	<0.001	0.02
FFM (kg)	Trails		11	11	11
	MD (95% CI)		−1.13 (−2.04, −0.23)	−1.61 (−2.68, −0.36)	−0.49 (−0.92, −0.05)
	Heterogeneity	*I* ^2^	0	0	0
		*P*	1.00	0.95	0.95
	*P*		0.01	0.01	0.03
HC (cm)	Trails		10	10	10
	MD (95% CI)		−4.17 (−5.48, −2.87)	−4.10 (−5.28, −2.91)	0.01 (−0.64, 0.67)
	Heterogeneity	*I* ^2^	0	41	25
		*P*	0.66	0.08	0.22
	*P*		<0.001	<0.001	0.97

BF, body fat; BMI, body mass index; BW, body weight; CER, continuous energy restriction; CI, confidence interval; FFM, fat free mass; HC, hip circumference; IER, intermittent energy restriction; MD, mean difference.

### 3.3. Risk of bias among the selected articles

**FIGURE 2:**
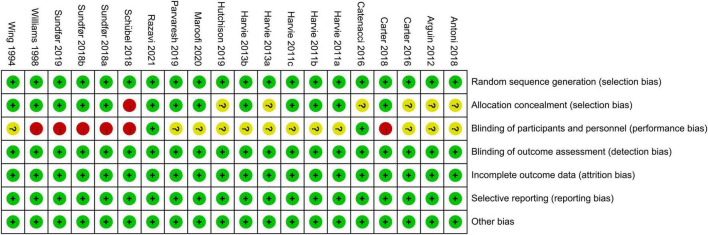
Risk of bias summary for the included studies.

**FIGURE 3:**
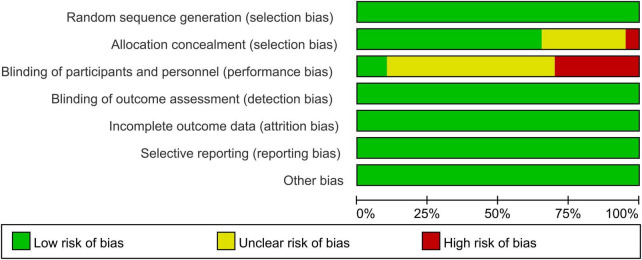
Risk of bias graph.

We assessed the 16 articles for risk of bias, and the results are shown in Figures 2, 3. In total, all studies had a low risk of bias for random sequence generation. One study’s authors (23) did not clearly state the allocation concealment, while 2 of the studies (37, 43) were double-blind experiments. All researchers completed the outcome data, and we found no other bias in the remaining studies.

### 3.4. Meta-analysis of overall effect

#### 3.4.1. Effect of IER and CER intervention on risk factors for MetS

**FIGURE 4:**
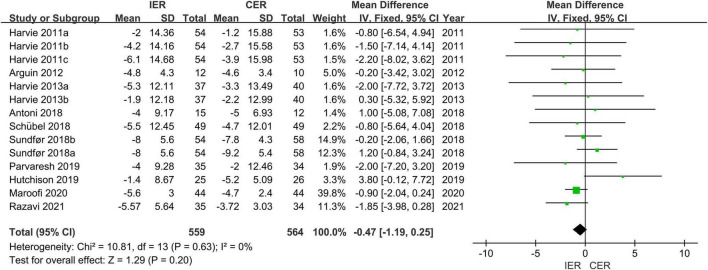
Effect of IER and CER intervention on WC.

Authors of 14 trials assessed WC, the results showed that a significant decrease in IER (MD = −5.34 cm, 95% CI [−6.71, −3.97] cm, *P* < 0.001, *I*^2^ = 0) and CER (MD = −5.02 cm, 95% CI [−6.34, −3.70] cm, *P* < 0.001, *I*^2^ = 0) after the diet interventions. When comparing IER and CER, there were no differences between groups in WC (MD = −0.47, 95% CI [−1.19, 0.25] cm, *P* = 0.20, *I*^2^ = 0) (Figure 4).

**FIGURE 5:**
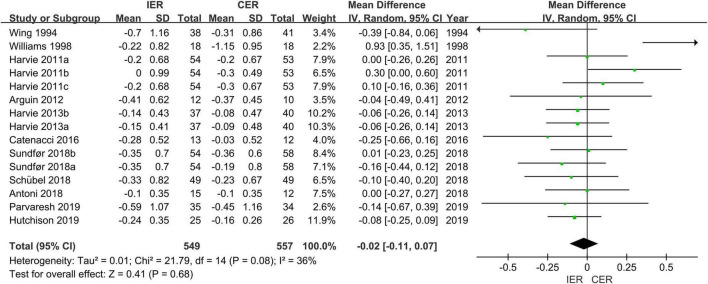
Effect of IER and CER intervention on TG.

Authors of 14 trials assessed TG, the results showed that a significant decrease in IER (MD = −0.21 mmol/L, 95% CI [−0.29, −0.13] mmol/L, *P* < 0.001, *I*^2^ = 0) and CER (MD = −0.21 mmol/L, 95% CI [−0.29, −0.14] mmol/L, *P* < 0.001, *I*^2^ = 0), but there was no difference between IER and CER (MD = −0.02 mmol/L, 95% CI [−0.11, 0.07] mmol/L, *P* = 0.08, *I*^2^ = 36%) (Figure 5).

**FIGURE 6:**
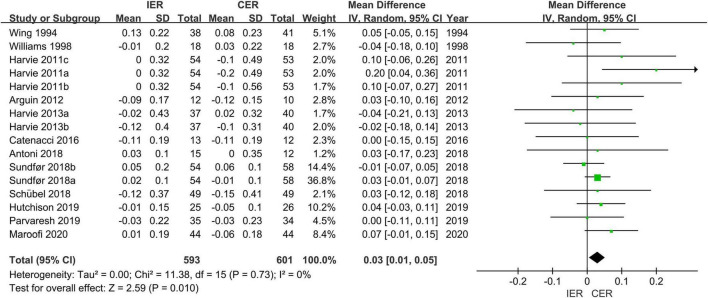
Effect of IER and CER intervention on HDL.

For HDL-c there was a significant improvement in IER (MD = 0.05 mmol/L, 95% CI [0.02, 0.08] mmol/L, *P* = 0.005, *I*^2^ = 24%) and CER (MD = 0.05 mmol/L, 95% CI [0.01, 0.08] mmol/L, *P* = 0.02, *I*^2^ = 11%), respectively, and an increase in IER compared to CER (MD = 0.03 mmol/L, 95% CI [0.01, 0.05] mmol/L, *P* = 0.02, *I*^2^ = 0) (Figure 6).

**FIGURE 7:**
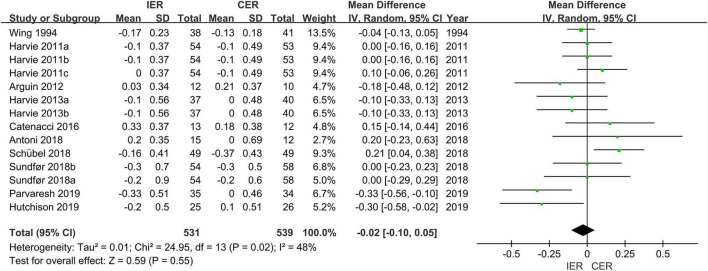
Effect of IER and CER intervention on FPG.

For FPG there was significant improvement in the IER group (MD = −0.07 mmol/L, 95% CI [−0.13, −0.01] mmol/L, *P* = 0.02, *I*^2^ = 43%), but not in the CER group (MD = −0.07 mmol/L, 95% CI [−0.17, 0.04] mmol/L, *P* = 0.22, *I*^2^ = 50%). There was also no difference between IER and CER (MD = −0.02 mmol/L, 95% CI [−0.10, 0.05] mmol/L, *P* = 0.73, *I*^2^ = 0) (Figure 7).

**FIGURE 8:**
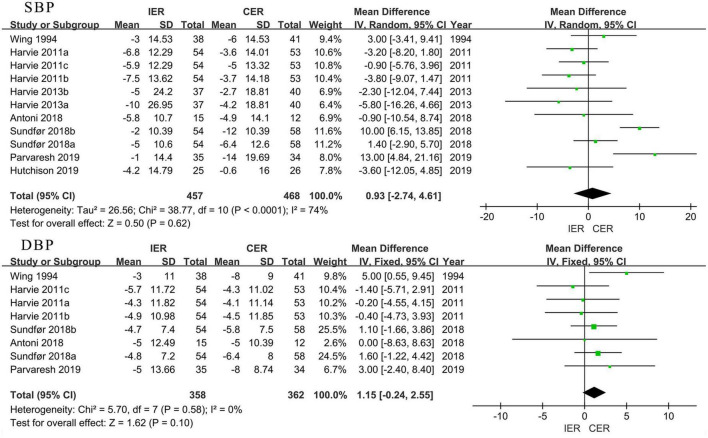
Effect of IER and CER intervention on BP.

Authors of 9 trials reported that systolic blood pressure (SBP) showed a significant decrease in IER (MD = −5.58 mmHg, 95% CI [−7.57, −3.6] mmHg, *P* < 0.001, *I*^2^ = 3%) and CER (MD = −5.59 mmHg, 95% CI [−7.52, −3.67] mmHg, *P* < 0.001, *I*^2^ = 0) after the diet intervention, but there was no difference between groups (MD = 0.93 mmHg, 95% CI [−2.74, 4.61] mmHg, *P* = 0.62, *I*^2^ = 74%). Authors of 7 trials reported a decrease in diastolic blood pressure (DBP) in both IER (MD = −5.67 mmHg, 95% CI [−7.11, −4.22] mmHg, *P* < 0.001, *I*^2^ = 0) and CER (MD = −4.87 mmHg, 95% CI [−6.48, −3.25] mmHg, *P* < 0.001, *I*^2^ = 0); authors of 8 trials compared the changes between IER and CER. The results showed no difference between the groups (MD =1.15 mmHg, 95% CI [−0.24, 2.55] mmHg, *P* = 0.10, *I*^2^ = 0) (Figure 8).

#### 3.4.2. Effect of IER and CER intervention on anthropometric index

Authors of 20 trials reported that BW has a significant decrease in IER and CER after diet intervention. Eight trials showed significant decrease in terms of BMI, while 11 trials showed significant decrease in terms of BF and FFM. When comparing IER and CER, BW (MD = −0.8 kg, 95% CI [−1.26, −0.33], *P* < 0.001, *I*^2^ = 0), BF (MD = −0.75 kg, 95% CI [−1.73, −0.13] kg, *P* = 0.02, *I*^2^ = 0) and FFM (MD = −0.49 kg, 95% CI [−0.92, −0.05] kg, *P* = 0.03, *I*^2^ = 0) were significantly decreased in the IER, but there were no significant changes in BMI (Table 2).

Authors of 10 trials assessed HC, the result showed a significant decrease in IER and CER after the diet interventions. When comparing IER and CER, there were no differences between groups in WC (MD = −0.47, 95% CI [−1.19, 0.25] cm, *P* = 0.2, *I*^2^ = 0) and HC (MD = 0.01, 95% CI [−0.64, 0.67] cm, *P* = 0.97, *I*^2^ = 25%) (Table 2).

#### 3.4.3. Effect of IER and CER intervention on blood index

Fourteen trials included TC and LDL-c, and both IER and CER showed a significantly decrease after intervention (*P* < 0.001). In 16 trials we compared the changes in TC and LDL-c between IER and CER before and after the intervention and found no difference between IER and CER in TC (MD = −0.04 mmol/L, 95% CI [−0.13, 0.04] mmol/L, *P* = 0.34, *I*^2^ = 0) and LDL-c (MD = −0.03 mmol/L, 95% CI [−0.11, −0.04] mmol/L, *P* = 0.37, *I*^2^ = 0) (Table 3).

**TABLE 3:** Comparison the effects of IER and CER on blood index.

Outcomes			Within-group		IER vs. CER
			**IER**	**CER**	
TC (mmol/L)	Trails		14	14	16
	MD (95% CI)		−0.36 (−0.48, −0.23)	−0.31 (−0.42, −0.21)	−0.04 (−0.13, 0.04)
	Heterogeneity	*I* ^2^	0	0	0
		*P*	0.48	0.88	0.54
	*P*		<0.001	<0.001	0.34
LDL-c (mmol/L)	Trails		14	14	16
	MD (95% CI)		−0.21 (−0.28, −0.14)	−0.17 (−0.27, −0.08)	−0.03 (−0.11, 0.04)
	Heterogeneity	*I* ^2^	0	0	0
		*P*	0.99	0.98	0.63
	*P*		<0.001	<0.001	0.37
FINs (μmol/L)	Trails		9	9	13
	MD (95% CI)		−0.33 (−0.48, −0.18)	−0.32 (−0.47, −0.17)	−0.08 (−0.66, 0.50)
	Heterogeneity	*I* ^2^	0	53	40
		*P*	0.50	0.03	0.07
	*P*		<0.001	0.005	0.78

CER, continuous energy restriction; CI, confidence interval; HDL-c, high-density lipoprotein cholesterol; IER, intermittent energy restriction; LDL-c, low-density lipoprotein cholesterol; MD, mean difference; TC, total cholesterol; TG, triglycerides.

Authors of 9 trials reported a change in FINs before and after the intervention. There was a significant decrease in both IER (MD = −0.33 μmol/L, 95% CI [−0.48, −0.18] mmol/L, *P* < 0.001, *I*^2^ = 0) and CER (MD = −0.32 mmol/L, 95% CI [−0.47, −0.17] mmol/L, *P* = 0.005, *I*^2^ = 53). Authors of 13 trials compared the change in FINs between IER and CER; there were no differences between IER and CER (MD = −0.08 mmol/L, 95% CI [−0.66, −0.5] mmol/L, *P* = 0.78, *I*^2^ = 40) (Table 3).

### 3.5. Sub-group analysis of the outcomes

Owing to the high heterogeneity, we performed subgroup analysis for intervention duration (≤4, 4–12, and >12 weeks). We found more weight loss with longer intervention duration (−0.75 vs. −1.12 kg), but not for intervention duration longer than 12 weeks. This could mean that IER reduced more BW than CER when the intervention duration was less than 12 weeks, but there was no significant difference between IER and CER when the intervention duration was longer than 12 weeks. We found a similar trend for FFM (Table 4).

**TABLE 4:** Sub-group analysis of the outcome.

Outcomes	Sub-group (weeks)	Trials	MD (95% CI)	Heterogeneity		*P*
				***I***^2^ **(%)**	***P***	
BW	≤4	7	−0.75 (−1.35, −0.16)	0	0.89	0.01
	≤12, >4	8	−1.12 (−2.14, −0.09)	0	0.70	0.03
	>12	5	−0.53 (−1.35, 0.29)	16	0.31	0.20
BMI (kg/m^2^)	≤4	2	−0.03 (−0.92, 0.85)	39	0.20	0.94
	≤12, >4	3	−0.60 (−1.32, 0.13)	0	0.62	0.11
	>12	3	−0.22 (−1.04, 0.61)	74	0.02	0.60
BF %	≤4	4	−0.15 (−0.68, 0.38)	0	0.97	0.59
	≤12, >4	2	0.37 (−0.83, 1.56)	0	0.84	0.55
	>12	2	−0.41 (−1.25, 0.44)	0	0.57	0.35
BF (kg)	≤4	4	−1.33 (−3.62, 0.96)	0	0.81	0.26
	≤12, >4	4	−0.28 (−1.75, 1.18)	11	0.34	0.70
	>12	2	−0.49 (−1.29, 0.31)	0	0.34	0.23
FFM (kg)	≤4	5	−0.63 (−1.30, 0.03)	0	0.96	0.06
	≤12, >4	2	−1.01 (−2.04, 0.01)	0	0.66	0.05
	>12	3	−0.08 (−0.78, 0.63)	0	0.98	0.83
WC (cm)	≤4	7	0.20 (−1.91, 2.31)	1	0.41	0.85
	≤12, >4	4	0.65 (−1.07, 2.36)	0	0.69	0.46
	>12	3	−0.80 (−2.08, 0.48)	0	0.48	0.22
HC (cm)	≤4	6	−0.39 (−1.33, 0.55)	52	0.06	0.42
	≤12, >4	4	0.39 (−0.52, 1.31)	0	0.98	0.40
	>12	2	0.12 (−1.66, 1.89)	49	0.16	0.90
TC (mmol/L)	≤4	7	0 (−0.19, 0.18)	49	0.07	0.97
	≤12, >4	6	−0.05 (−0.23, 0.12)	0	0.91	0.53
	>12	3	−0.04 (−0.22, 0.14)	0	0.65	0.63
TG (mmol/L)	≤4	6	0.01 (−0.10, 0.12)	17	0.30	0.87
	≤12, >4	6	−0.11 (−0.23, 0.01)	0	0.71	0.07
	>12	2	0 (−0.21, 0.21)	0	0.85	0.99
HDL-c (mmol/L)	≤4	6	0.07 (0.01, 0.12)	12	0.34	0.02
	≤12, >4	7	0.02 (−0.01, 0.05)	0	0.90	0.17
	>12	2	0 (−0.10, 0.09)	0	0.47	0.93
LDL-c (mmol/L)	≤4	7	−0.05 (−0.17, 0.06)	30	0.20	0.36
	≤12, >4	6	−0.04 (−0.16, 0.09)	0	0.65	0.56
	>12	3	0.01 (−0.15, 0.17)	0	0.84	0.92
FPG (mmol/L)	≤4	6	−0.08 (−0.21, 0.05)	61	0.02	0.22
	≤12, >4	6	0.05 (−0.07, 0.16)	45	0.10	0.42
	>12	2	−0.07 (−0.25, 0.12)	0	0.35	0.48
FINs (pmol/L)	≤4	7	−0.58 (−1.13, −0.03)	14	0.32	0.04
	≤12, >4	5	0.97 (−0.01, 1.95)	0	0.60	0.05
SBP (mmHg)	≤4	6	−0.51 (−4.87, 3.85)	63	0.02	0.82
	≤12, >4	4	0.87 (−2.23, 4.06)	0	0.54	0.59
DBP (mmHg)	≤4	4	−0.02 (−2.29, 2.25)	0	0.65	0.99
	≤12, >4	3	2.39 (0.10, 4.68)	0	0.38	0.04

BF, body fat; BF (%), body fat percentage; BMI, body mass index; BW, body weight; CER, continuous energy restriction; CI, confidence interval; DBP, diastolic blood pressure; FFM, fat free mass; FINs, fasting insulin; FPG, fasting plasma glucose; HC, hip circumference; HDL-c, high-density lipoprotein cholesterol; IER, intermittent energy restriction; LDL-c, low-density lipoprotein cholesterol; MD, mean difference; SBP, systolic blood pressure; TC, total cholesterol; TG, triglycerides; WC, waist circumference.

For TGs, FIN, and DBP we found that the intervention effect of IER was significantly better than CER when the intervention duration was between 4 and 12 weeks, but there were no significant differences between the IER and CER groups when the duration was longer than 12 weeks. For other outcomes, the changes in IER were not more significant than the CER group owing to the longer intervention duration (Table 4).

## 4. Discussion

Our systematic review provides RCT-based evidence on the efficacy of continuous and intermittent energy restriction diet protocols on weight loss, body composition, blood pressure, and other cardiometabolic risk factors in overweight and obese individuals with MetS risk factors. IER and CER have been attracting attention as caloric restriction protocols for reducing energy intake and improving lipid metabolism in obesity, T2DM, and MetS. Though IER and CER are two popular protocols that attracted a lot of attention, but the effect on risk factors of MetS has been inconclusive.

The current meta-analysis included 16 studies and 1,511 participants. The common diagnostic criteria for MetS included BW, BMI, body composition, WC and HC, lipid profile, glycemic measures, and BP (44). This review revealed that BW, BMI, body composition, WC and HC, TC, TG, FINs, SBP, and DBP showed a significant decrease, and HDL-c showed a significant increase in both IER and CER.

In this study we found that IER is more effective than CER in reducing BW and FFM. Decreased BW and body fat are important prerequisites for improving lipid metabolism disorders and alleviating MetS biomarkers (45), but prolonged calorie restriction downregulates skeletal muscle protein synthesis (46). Weight loss is frequently accompanied by a reduction in lean body mass and muscle mass (47, 48). Therefore, FFM was significantly reduced in this meta-analysis. Wang et al. (26) observed that intermittent fasting is more effective in achieving weight loss and FFM loss in patients with T2DM and MetS than a continuous energy-restricted diet. Since it is fat mass loss that improves health indices and not the loss of muscle, the significantly greater loss of lean mass is concerning and needs to be further assessed. However, there was no significant difference in WC and HC between IER and CER in this meta-analysis. Seimon et al. reported similar results (49).

Andrea et al. (25) found that the duration of intervention is not enough to conclude that one intervention is more effective than the other. In this meta-analysis the subgroup analysis indicated that reductions in weight loss in IER were apparently greater than in CER in short- (≤4 weeks) and medium-term (< 12, > 4 weeks) interventions. We obtained similar results for FFM loss. Overall, IER appeared to be more effective than CER in BW management. However, when the intervention period was extended, any difference in the effectiveness between IER and CER disappeared.

Evidence increasingly suggests that modest and sustained weight loss improved glycemic control in overweight and obese individuals and induced decreases in pancreatic and liver TG level (50). The lipid profiles and glycemic measures could reflect glycolipid metabolism at a deeper level. There is essentially no difference between obesity and MetS in body composition and WC and HC. However, glycolipid metabolism disorders have already occurred in patients with MetS. In this meta-analysis, without considering the fasting regime only IER significantly reduced FPG after intervention, but there was no significant difference in the improvement of FPG and FINs between IER and CER. Wang et al. (26) also reported no differences between IER and CER regimens in their data on FINs. For FINs, it seems that the effect size was significantly improved by IER (MD = −0.08 mmol/L, 95% CI [−0.66, −0.5] mmol/L, *P* = 0.78), but the heterogeneity was moderate (*I*^2^=50%), this may be due to some of the studies included was T2DM participants. Additionally, IER was effective than CER in improving HDL-c (MD = 0.03 mmol/L, 95% CI [0.01, 0.05] mmol/L, *P* = 0.02, *I*^2^ = 0). Both TG and HDL-c are the major parameters of verifying MetS (29), lower HDL-c levels are commonly accompanied with an increase in TG-rich lipoproteins in obesity (51), and the reduction in HDL-c would affect lipid metabolism in obesity or insulin resistance (52).

Researchers have reported that dyslipidemia, especially hypertriglyceridemia, is an independent risk factor in predicting the development of diabetes, which is partially mediated by insulin resistance and obesity (53). Some authors suggest that HDL-c did not significantly change in intermittent or continuous energy restriction (26, 53). Meng et al. (53) included healthy and obese individuals in their meta-analysis, while Wang et al. (26) included only 3 trials in their study. According to sub-analysis, the results of HDL-c showed that when intervention duration less than 4 weeks, IER was significantly better than CER. Mechanistically, even if the total energy deficit was consistent in each week, IER could cause a larger energy deficit on fasting days and more effective fat oxidation. Previous studies suggested that IER has been shown to be more effective than CER in improving lipid metabolism, resulting in a greater loss of BF following the IER regimen (16, 18). Two studies of overweight and obese women suggested greater reductions in insulin concentrations and fat mass with IER than with CER over 4 and 6 months, with similar figures for net calorie intake and weight loss (11, 24). As shown in this meta-analysis, IER strategies were more effective than CER in improving HDL-c, but not for other risk factors for MetS (WC, TG, FPG, BP). Further research should expand the number of subjects or take gender difference into account to explore the differences between IER and CER.

In conclusion, both IER and CER could improve MetS biomarkers. IER was more effective than CER in the criteria of BW, FFM, TGs, HDL-c, and FPG, but no difference for more than 12 weeks. Although the data are insufficient, our study shows that IER is superior to CER in patients with MetS risk factors, and the differences between IER and CER in the short- and medium-term intervention periods could be more remarkable. Hence, future studies are needed to investigate the differences in the long-term intervention effects of IER and CER.

Maybe IER regimens should be tried in clinical practice, some individuals find easier to reduce energy intakes for 1 or more days per week, rather than every day. But it is essential that IER strategies should be considered by health professionals. It is well known that a single diet fit not all. A single dietary pattern is not suitable for everyone, and for people with metabolic syndrome, the right is the best.

## 5. Limitations

We acknowledge 3 limitations in our work. First, it was difficult to conduct double-blinded studies of dietary supervision because most of the studies used 7-day diet records or checklists to supervise and compile statistics on the participants’ caloric intake. The researchers provided the food during the interventions only in the studies conducted by Catenacci et al. (37) and Hutchison et al. (22). Self-reported adherence to the fasting days was variable and had an influence on the results. Second, we did not explore the influence of IER and CER interventions on adherence, appetite, or adverse events. Third, there is limited evidence specifically focused on adults with sexual dimorphism. We did not analyze gender differences in subgroups, and as such it is unclear whether IER and CER interventions would produce the same results as we reported here when applied to males or females.

## Author contributions

RX contributed to study conception and design, drafting the submitted article, and critically revising the draft for important intellectual content. RX, YC, X-LC, P-YW, and DT contributed to acquisition, analysis, and interpretation the data. All authors contributed at all stages of this study, gave final approval of the version for publication, and agreed to be accountable for all aspects of the work.
