# Supplementary material for: Intermittent energy restriction vs. continuous energy restriction on cardiometabolic risk factors in patients with metabolic syndrome: a meta-analysis and systematic review
Source: Front Nutr. 2023 May 9;10:1090792. doi: 10.3389/fnut.2023.1090792 (PMC10204925; doi:10.3389/fnut.2023.1090792)
Supplement: Supplementary file 1 [file Table_1.DOCX]

**Table 1** Complete literature search

| MEDLINE/PubMed | |
| --- | --- |
| #1 | (((((((Intermittent fasting) OR (Intermittent Energy Restriction)) OR (Alternate day fasting)) OR (Periodic fasting)) OR (time-restricted feeding)) OR (intermittent caloric restriction)) AND ((((obesity[MeSH Terms]) OR (obes*[Title/Abstract])) OR (overweight[Title/Abstract])) OR (adiposity[Title/Abstract]))) AND ((((metabolic syndrome x[MeSH Terms]) OR (Metabolic Syndrome[Text Word])) OR (Syndrome X[Text Word]))) |
| #2 | (((((((Intermittent fasting) OR (Intermittent Energy Restriction)) OR (Alternate day fasting)) OR (Periodic fasting)) OR (time-restricted feeding)) OR (intermittent caloric restriction)) AND ((((obesity[MeSH Terms]) OR (obes*[Title/Abstract])) OR (overweight[Title/Abstract])) OR (adiposity[Title/Abstract]))) AND ((((metabolic syndrome x[MeSH Terms]) OR (Metabolic Syndrome[Text Word])) OR (Syndrome X[Text Word]))) |
| #3 | (((metabolic syndrome x[MeSH Terms]) OR (Metabolic Syndrome[Text Word])) OR (Syndrome X[Text Word])) |
| #4 | (((obesity[MeSH Terms]) OR (obes*[Title/Abstract])) OR (overweight[Title/Abstract])) OR (adiposity[Title/Abstract]) |
| #5 | (((((Intermittent fasting) OR (Intermittent Energy Restriction)) OR (Alternate day fasting)) OR (Periodic fasting)) OR (time-restricted feeding)) OR (intermittent caloric restriction) |
| EMBASE | |
| #1 | 'intermittent fasting'/exp |
| #2 | 'fasting'/exp OR 'energy restriction'/exp OR 'caloric restriction'/exp |
| #3 | 'metabolic syndrome x'/exp |
| #4 | 'obesity'/exp |
| #5 | #1 OR #2 |
| #6 | #3 AND #4 AND #5 |
| #7 | 'randomized controlled trial'/exp |
| #8 | #6 AND #7 |
| The Cochrane Library | |
| #1 | MeSH descriptor: [Fasting] explode all trees |
| #2 | Alternate day fasting |
| #3 | Intermittent energy restriction |
| #4 | Alternate day fasting |
| #5 | Periodic fasting |
| #6 | time-restricted feeding |
| #7 | intermittent caloric restriction |
| #8 | MeSH descriptor: [Metabolic Syndrome] explode all trees |
| #9 | #1 OR #2 OR #3 OR #4 OR #5 OR #6 OR #7 |
| #10 | #8 AND #9 |
| SPORTDiscus | |
| #1 | ( intermittent fasting and time restricted feeding and time restricted eating or intermittent caloric restriction ) AND ( metabolic syndrome or metabolic disease or syndrome x ) AND ( obese or obesity or overweight ) |
